# The Role of Salivary Microbiota in Pancreatic Cancer: From Screening to Tumor Progression and Treatment Response

**DOI:** 10.3390/biomedicines14061407

**Published:** 2026-06-22

**Authors:** Marco Donatello Delcuratolo, Giovanna Cocomazzi, Viria Beccia, Concetta Panebianco, Elena Binda, Valerio Pazienza, Tiziana Pia Latiano

**Affiliations:** 1 Oncology Unit, Fondazione IRCCS Casa Sollievo Della Sofferenza, 71013 San Giovanni Rotondo, FG, Italy; v.beccia@operapadrepio.it (V.B.); t.latiano@operapadrepio.it (T.P.L.); 2 Microbiome and Pharmacomicrobiomics Unit, Fondazione IRCCS Casa Sollievo Della Sofferenza, 71013 San Giovanni Rotondo, FG, Italy; g.cocomazzi@operapadrepio.it (G.C.); c.panebianco@operapadrepio.it (C.P.); v.pazienza@operapadrepio.it (V.P.); 3 Cancer Stem Cell Biology Research Unit, Fondazione IRCCS Casa Sollievo Della Sofferenza, 71013 San Giovanni Rotondo, FG, Italy; e.binda@operapadrepio.it

**Keywords:** microbiota, saliva, pancreatic cancer, cancer detection, prognosis

## Abstract

Pancreatic cancer (PC) remains one of the malignancies with the most unfavorable prognosis and limited treatment options. The lack of biomarkers for early diagnosis and the asymptomatic nature of the disease contribute to delays in diagnosis and high mortality rates. In recent years, the role of the human microbiota in cancer biology has become increasingly significant, and the oral microbiota in particular has been found to be involved in the pathogenesis and prognosis of several neoplasms. This review summarizes the current evidence relating the salivary microbiota to PC in three key areas: screening and diagnostic potential, pathophysiology and tumor progression, as well as presenting prognostic implications and potential influence on therapy. With regard to early diagnosis, it has been reported that patients with PC have reduced levels of *Neisseria elongata* (*N. elongata*) and *Streptococcus mitis* (*S. mitis*) and elevated levels of *Granulicatella adiacens*. Several studies have shown that bacteria present in the saliva can migrate from the oral cavity to pancreatic tissue via hematogenous or enteric routes, where they may actively contribute to tumor development and progression. In particular, it has been shown that *Porphyromonas gingivalis* (*P. gingivalis*) and *Veillonella atypica* (*V. atypica*) translocate from the mouth to pancreatic tumors, promoting carcinogenesis by inducing a pro-inflammatory tumor microenvironment. Furthermore, some studies have identified certain species associated with prognosis and response to PC treatment. Despite the encouraging results, differences in study methodology, the lack of standardized methods and the scarcity of longitudinal data currently hinder clinical application. Large-scale, multi-omics prospective studies are needed to clarify causality and validate their clinical utility. Overall, the salivary microbiota represents a promising and non-invasive tool for improving early diagnosis, understanding prognosis and enhancing the management of PC.

## 1. Introduction

Pancreatic cancer (PC) is classified among the most lethal malignancies worldwide, characterized by a dismal prognosis and limited therapeutic options. Despite significant advances in diagnostic and treatment intervention, PC displays a 5-year survival rate of below 10% [1]. Pancreatic ductal adenocarcinoma (PDAC) is the most common type, accounting for over 90% of all pancreatic cancers. It is often diagnosed at an advanced stage due to its asymptomatic nature during onset, without any reliable early disease biomarker, reducing the chance of curability [2]. Most (80–90%) patients diagnosed with PC are incurable, and only a small percentage of patients are eligible for surgical resection, which is currently the only curative treatment available [3]. The epidemiology of PC is complex and multifactorial. Risk factors, most of which are shared with other types of cancer, include age, smoking, obesity, chronic pancreatitis, diabetes mellitus, family history, and specific genetic mutations [4,5]. Family history and genetic mutations are important risk factors. Having two or more first-degree relatives with PDAC increases the risk of developing PC by 6.79 times [6]. Mutations in the Breast Cancer Type 2 Susceptibility Gene (*BRCA2*) and Cyclin-Dependent Kinase Inhibitor 2A (*CDKN2A*) gene are responsible for most cases of familial PDAC. In addition, the risk of developing PDAC is elevated in patients with hereditary diseases, such as Lynch syndrome with MutS Homolog 2 (*MSH2*) and MutL Homolog 1 (*MLH1*) mutations, familial adenomatous polyposis (Adenomatous Polyposis Coli, *APC* mutation), Peutz–Jeghers syndrome (Serine/Threonine Kinase 11, *STK11*/Liver Kinase B1, *LKB1* mutation), and familial atypical multiple melanoma syndrome (*CDKN2A*/*P16* mutation) [7,8]. The abovementioned risk factors, however, only partially explain the disease etiology, making the exploration of additional environmental and biological contributors necessary.

Although carbohydrate antigen 19-9 (CA19-9) remains the most widely used serum biomarker, its diagnostic effectiveness is suboptimal, particularly in the early stages of the disease. Consequently, there is a growing need to identify innovative and minimally invasive biomarkers capable of improving risk stratification, early diagnosis, prognostic assessment and therapeutic monitoring.

In recent years, increasing attention has been given to the role of the human microbiome in modulating cancer risk, including that of PC. The human ecosystem is made of more prokaryotic organisms than eukaryotic cells. In fact, only one out of 10 cells in our bodies is human [9]. These commensal microorganisms colonize not only the human gastrointestinal tract but also the mouth, the respiratory tract, the skin, and other anatomic sites [10]. Accumulating evidence suggests that a disruption to the microbial ecology is involved in cancer biology through the induction of inflammation, or the modulation of host immune functions. Dysbiosis, or an imbalance in the composition of microbial communities, has been implicated in various chronic inflammatory diseases and cancers [11]. Moreover, the production of specific metabolites has been proposed as a potential mechanism to explain how microorganisms influence cancer development [12]. The gut microbiota may play a role in the onset and progression of PC. Recent studies demonstrate that the gut microbiota of PC patients is different from that of healthy people, with a reduction in short-chain fatty acid (SCFA) producers *Firmicutes* and *Lactobacillus* and an increase in potentially pathogenic species such as *Proteobacteria*, *Porphyromonas*, *Prevotella*, and *Helicobacter pylori* [13,14,15,16,17]. In the external wall of Gram-negative bacteria, lipopolysaccharide (LPS) acts as a potent endotoxin capable of activating an inflammatory response through the production of tumor necrosis factor alpha (TNF-α) and the interleukins IL-6 and IL-1, causing inflammatory damage [18]. Furthermore, it has been shown that mice expressing oncogenic Ras exposed to LPS developed chronic pancreatitis and Pancreatic Intraepithelial Neoplasia (PanIN) lesions [19]. The pancreas is not a sterile organ, as demonstrated by the presence of bacteria in pancreatic tissue, such as *Enterococcus faecalis* and *Escherichia coli* [15]. In particular, the high presence of *Enterococcus faecalis* and the high antibody titer against the capsular polysaccharide of *Enterococcus faecalis* in the pancreatic tissue of patients with chronic pancreatitis and PC demonstrate that this microorganism plays a role in the inflammation and carcinogenesis of PC [20]. Although scientists focus primarily on the gut microbiota, where most microorganisms reside, the oral cavity also harbors a diverse microbial ecosystem. Recent studies have investigated the human oral microbiota and its contribution to the development and progression of cancer. The salivary microbiota of healthy individuals is mainly represented by six broad phyla: *Firmicutes*, *Actinobacteria*, *Proteobacteria*, *Fusobacteria*, *Bacteroidetes* and *Spirochaetes* [21]. Oral pathogens may influence pancreatic carcinogenesis through both direct and indirect mechanisms, including systemic inflammation, immune modulation, and the translocation of microbial components to distant organs [22,23]. Plausible biological mechanisms have been proposed to explain the relationship between the oral microbiota and PC. Pushalkar et al. hypothesized that oral pathogens or their metabolites can reach the pancreas via hematogenous or enteric routes, triggering local inflammation or altering the pancreatic microenvironment favoring tumor development [15]. Considering the high mortality associated with PC and the urgent need for early detection strategies and efficient treatment options, further exploration into the role of the oral microbiota is warranted. A better understanding of the microbial signatures and their functional roles could lead to novel diagnostic tools and potentially modifiable risk factors. This manuscript aims to review the evidence linking salivary microbiota to pancreatic carcinogenesis, with a particular focus on its potential roles in screening and diagnostic strategies, tumor pathophysiology and progression, and its prognosis and influence on therapeutic response. We highlight key knowledge gaps and future research directions that may contribute to the development of more effective prevention and early detection strategies for this fatal disease.

## 2. Oral Microbiota

**Figure 1:**
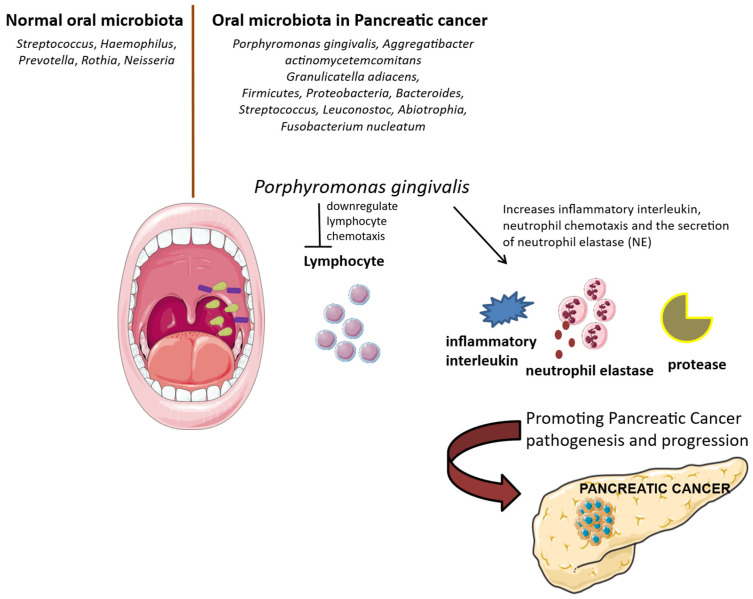
Oral microbiota in healthy individuals and pancreatic cancer patients. The normal microbiota is dominated by *Streptococcus*, *Haemophilus*, *Prevotella*, *Rothia* and *Neisseria* and is maintained by saliva, preventing the implantation of pathogenic species. The oral microbiota in pancreatic cancer is dominated by pathogenic species such as *P. gingivalis*, which reduces lymphocyte chemotaxis, preventing the defense mechanism against pathogens while increasing the expression of genes associated with the inflammatory response by increasing the release of inflammatory interleukins; in addition, *P. gingivalis* increases the secretion of neutrophil elastase (NE) from neutrophils and proteases to promote the progression of pancreatic cancer.

The oral microbiome, the second largest microbiome following the gut microbiome, houses over 700 species of bacteria, fungi, viruses, and protozoa. Different species grow and colonize the oral cavity, evolving with the host and competing with the other species, rapidly adapting to changes in internal and external conditions. These different species are organized in specified microenvironments with a role in metabolic exchange with local and systemic immunogenic interactions, affecting the oral cavity but also systemic health. Cell-to-cell adhesion between bacterial species leads to colonization and the formation of a structured polymicrobial community architecture. Bacterial species compete, at spatial and metabolic levels, with other species, through direct contact, peptide-induced internal signaling cascade, modulation of gene expression, substrate availability and limited space using compounds such as bacteriocin and H_2_O_2_, which play a crucial role in defining the structure and activity of oral microbial communities. So, the ecological equilibrium of this well-organized, multispecies oral community is maintained through competitive and cooperative interactions between microorganisms of the same or different species at the cellular and molecular levels [24]. Despite this challenging ecosystem, Belstrom et al. have demonstrated that the oral microbiome remains substantially stable through time [25]. The different anatomical niches lead to the definition to three main microbiotas: dental plaque, the tongue dorsum and the keratinized gingiva, where different species of microorganism can be detected in their true relative abundance. For example, genera such as *Fusobacterium* and *Veillonella*, and families such as the *Prevotellaceae*, contain separate, distinct species that are specialized for the tongue, dental plaque or gums. Moreover, because of microorganism shedding into saliva, which is distributed throughout the mouth, most oral microorganisms are detectable at any oral site and salivary microbiota is the most easily studied [26,27]. Oral *Streptococci* are believed to be among the earliest inhabitants on tooth surfaces due to their ability to adhere directly to salivary pellicle and comprise about 80% of the early colonizers. Other early colonizers are *Actinomyces*, *Veillonella* and *Neisseria* [9]. Sakanaka et al. have demonstrated that interactions between *Fusobacterium nucleatum* (*F. nucleatum*) and early-colonizing commensals can influence biofilm development and the dispersion of later-colonizing pathogens, such as *Porphyromonas gingivalis* (*P. gingivalis*) [28]. The importance of communication between species, not only bacterial, is exemplified by the presence of *Candida albicans* (*C. albicans*). The interactions between *C. albicans* and *Streptococcus mutans* or *Streptococcus oralis* exacerbate the severity of oral candidiasis or dental caries, respectively. Meanwhile, cooperation between *C. albicans* and *F. nucleatum* is capable of evading the host immune system [29]. Baker et al. underscore that the complexity of interactions between species requires a holistic approach. Recent technologies can better appreciate and study this enormous complexity, without reducing oral microbiome to the study of individual, more abundant bacteria [29]. Due to the complex competitive interactions between species and the tight interactions between the microorganism and their host, through regulation of the immune system, oral microbiota is linked to major oral pathologies, such as dental caries and periodontal disease. Caries is associated with a dysbiosis of the dental plaque microbiota, while periodontal disease is an inflammatory disruption in the host–microbial homeostasis of the periodontal pocket. Pathologies can cause disruptions in the oral microbiome. But the opposite is also true. Periodontitis causes local tissue disruption through a dysregulated activation of the inflammatory response and the outgrowth of pathogens. Moreover, microorganisms such as *P. gingivalis*, *Aggregatibacter actinomycetemcomitans* (*A. actinomycetemcomitans*) and *F. nucleatum* have been linked to several systemic chronic diseases, including Alzheimer disease, diabetes, cardiovascular disease, inflammatory bowel disease, rheumatoid arthritis, nonalcoholic fatty liver disease and obesity [30]. In addition, oral dysbiosis may promote carcinogenesis through a variety of interconnected mechanisms, including sustained chronic inflammation, immune dysregulation, the production of carcinogenic metabolites, the activation of oncogenic signaling pathways, and the spread of microorganisms or their products to distant tissues, thus promoting the onset and progression of cancer [31]. Several studies have highlighted the association between oral microbiome alterations and head and neck, pancreatic, colorectal, and lung cancers. The mechanisms involved are related to the translocation and dissemination of oral bacteria via hematogenous translocation (transient bacteremia), through digestive tract pathways, or through triggering an inflammatory response and consequently increasing cancer risk [32] (Figure 1).

According to a recent review, *P. gingivalis*, *Fusobacterium*, *N. elongata* and *S. mitis* are the main pathogens correlated with PC [33]. The cohort study of Meng et al. demonstrated that, beyond the assured role of *P. gingivalis*, *Eubacterium nodatum* and *Parvimonas micra*, which are oral bacterial periodontal pathogens, are also associated with increased risk of PC [34]. Saba et al. demonstrated that *P. gingivalis* translocates from the oral cavity to the pancreas and promotes tumor cell proliferation, even in the absence of oral inflammation [35]. *P. gingivalis* can influence eukaryotic cell cycle through changing onco-suppressor phosphorylation and inhibits apoptosis through protein rearrangement [36]. Binder Gallimidi et al. reported that *P. gingivalis* and *F. nucleatum* both induce tumorigenesis through enhanced production of inflammatory interleukin and protease [37]. Ahn et al. prospectively examined the association between *P. gingivalis* and mortality from orodigestive cancer, even without the presence of periodontitis [38].

The major correlations between salivary microbiota and PC will be thoroughly discussed in the subsequent paragraphs.

## 3. Methods

This narrative review aims to provide a comprehensive overview of the role of the salivary microbiota in pancreatic cancer. In particular, it focuses on its involvement in screening and diagnosis, tumor pathophysiology, and progression, as well as its prognostic implications and influence on treatment strategies. A non-systematic literature search was conducted using the PubMed database. The following search terms were applied: “oral microbiota pancreatic cancer” and “salivary microbiota pancreatic cancer”. Only original research articles explicitly investigating the salivary microbiota (salivary samples) in the context of pancreatic cancer were considered eligible.

## 4. The Role of Salivary Microbiota in Pancreatic Cancer

The saliva test for microbiota analysis is considered simpler and more acceptable to patients than fecal collection. The oral microbiota is a collection of numerous and diverse microorganisms rich in species diversity [39]. The most common genera in the salivary microbiota of healthy individuals are: *Streptococcus*, *Haemophilus*, *Prevotella*, *Rothia* and *Neisseria* [25]. The balance of different bacterial species in the oral microbiota is maintained by saliva, which increases the concentration of bicarbonate and makes the pH of the oral cavity neutral, thus preventing the implantation of pathogenic species [40]. In addition, saliva contains mucins, secretory immunoglobulins, lactoferrin, lysozyme, and lactoperoxidase, which can perform antibacterial, antifungal, and bacteriostatic functions [41]. Understanding the infectious factors that contribute to PC requires an understanding of the oral and salivary microbiota.

### 4.1. Screening and Diagnostic Potential

The clinical value of analyzing the salivary microbiota in PC extends to both screening and diagnostic applications (Table 1). From a screening perspective, the aim is to identify patients with PC within otherwise healthy or asymptomatic individuals. From a diagnostic perspective, salivary microbial signatures may also help to distinguish PC from benign pancreatic diseases, such as chronic pancreatitis, and from precursor lesions, including PanIN and intraductal papillary mucinous neoplasms (IPMNs). In this context, several studies have investigated the potential role of the salivary microbiota as a non-invasive diagnostic biomarker. Farrell et al. conducted a study divided into various phases; in the first, using the Human Oral Microbe Identification Microarray (HOMIM), they identified 31 increased species/clusters that were increased in the saliva of 10 PC patients compared to healthy controls (*n* = 10), while 25 species/clusters were decreased. Specifically, 16 species/clusters reached statistical significance (*p* < 0.05) and were selected as biomarker candidates. Of these, six species were confirmed in quantitative Polymerase Chain Reaction (qPCR) and in a larger validation cohort, *N. elongata* and *S. mitis* were found to be reduced in PC patients (*p* < 0.05). Specifically, the combination of *N. elongata* and *S. mitis* yielded a sensitivity of 96.4% and a specificity of 82.1% in distinguishing PC patients from healthy subjects (*p* < 0.0001). In contrast, *Granulicatella adiacens* was found to be increased in patients with neoplasia and also allows for the differentiation of these from those with chronic pancreatitis (*p* < 0.05) [42]. These results overlap with those of the recent study by Tavanaeian et al., conducted on 20 patients with PC and 20 control cases. In the former, an increase in *Granulicatella adiacens* levels was recorded (*p* < 0.001), while *N. elongata* levels were lower than in healthy subjects (*p* < 0.001) [43]. Although the biological significance highlighted by these independent cohorts has not yet been established, *Neisseria* and other *Proteobacteria* are generally considered to be components of a healthy oral microbiota and contribute to the nitrate reduction pathways involved in oral homeostasis, whilst an increase in the abundance of *Granulicatella* has often been observed in conditions of dysbiosis and oral inflammation [44,45,46].

Another study evaluated the difference in salivary microbiota between untreated PC patients, IPMNs patients and healthy controls. No differences were found between the three groups on measures of the alpha diversity of the oral microbiota. Nevertheless, PC cases had higher levels of *Firmicutes* and related taxa, whereas controls had higher levels of *Proteobacteria* and related taxa; however, differences between PC and IPMNs cases were less marked [47]. The pilot study conducted by Lin et al. showed that *Bacteroides* genus was significantly more prevalent in both PC patients (*p* = 0.002) and pancreatitis patients (*p* = 0.004) compared with the control group. Furthermore, *Corynebacterium* (*p* = 0.033) and *Aggregatibacter* (*p* = 0.019) were less common in the PC and pancreatitis groups than in the controls [48]. The study by Torres et al. also showed a lower abundance of *Aggregatibacter*, as well as *Neisseria*, in PC patients, with a higher prevalence of *Bacteroides*; furthermore, a significantly higher ratio of *Leptotrichia* to *Porphyromonas* was identified in their saliva compared to both healthy individuals and those with other diseases (*p* < 0.001) [49]. Another experience conducted on 10 PC patients, 17 with pancreatitis and 10 healthy controls, showed the same trends as the studies mentioned above. In particular, at the phylum level, patients with neoplasia had higher percentages of *Bacteroidetes*, *Firmicutes* and *Fusobacteria*, while the percentages of *Proteobacteria* were lower than in healthy individuals. Notably, the higher abundance of *Fusobacterium periodonticum* and the lower abundance of *Neisseria mucosa* were identified as potential microbial risk factors for PC. Furthermore, eight pathways demonstrated significant changes when comparing the PC and healthy groups and the pancreatitis and healthy groups (*p* < 0.05), including four pathways related to glucose metabolism [50]. In contrast, the prospective study by Fan et al. showed that the saliva abundance of *Fusobacteria* was associated with reduced risk of PC (OR 0.94) as well as its genus *Leptotrichia* (OR 0.87); conversely, both *P. gingivalis* and *A. actinomycetemcomitans* were associated with increased risk of PC (OR 1.60 and OR 2.20, respectively) [22]. Hesami et al. confirmed the increased risk of PC associated with high levels of *P. gingivalis*, particularly in females, and of *A. actinomycetemcomitans*, particularly in diabetic individuals. Furthermore, a model based on these two species associated with diabetes and circulating levels of miR-21 and miR-155 was developed, achieving a sensitivity of 87.8% and a specificity of 78.9% [51]. As some experiences mentioned [43,48,49], an Iranian case–control study identified low levels of *Proteobacteria* in PC. In fact, by collecting salivary samples from 273 PC cases and 285 controls, *Haemophilus* and related higher taxa (*Proteobacteria*, *Gammaproteobacteria*, *Pasteurellales*, and *Pasteurellaceae*) were found to be associated with lower odds ratios of neoplasia (OR 0.95). In contrast, the presence of *Enterobacteriaceae* (OR 2.8), *Lachnospiraceae G7* (OR 2.4), *Bacteroidaceae* (OR 1.90), and *Staphylococcaceae* (OR 1.81) was correlated with a higher odds ratio of PC [52]. A prospective study that collected 110 saliva samples (41 from PC patients and 69 from healthy individuals) identified *Neisseria* (OR = 0.309) and *Veillonella* (OR = 0.187) as potentially associated with a lower risk of PC; in contrast, *Streptococcus* and *Leptotrichina* were found to be associated with an increased risk of PC (OR 5.344 and OR 6.886, respectively). In addition, *Escherichia coli* and *Tannerella forstythia* correlated with a significant increase in unresectable PC. The authors also reported that PC patients showed significantly higher microbial abundance but lower microbial diversity in their oral microbiota [53]. A recent case–control study evaluated the salivary microbiome from gastric (GC), esophageal (EC), biliary (BC) and PC patients compared to age- and sex-matched healthy controls. Although no microbial taxa with diagnostic value were identified for both BC and PC, the authors reported a difference in microbiome composition between EC vs. PC (*p* = 0.001), GC vs. PC (*p* = 0.001) and BC vs. PC (*p* = 0.013), supporting the idea that oral dysbiosis is more strongly associated with tumors closer to the oral cavity. Furthermore, in agreement with other studies [22,42,43,47,48,49,52], *Neisseria* abundance was decreased in PC patients compared to healthy individuals [54]. A recent study by Uguz et al. compared the salivary and tissue microbiota of 20 patients with PC, 10 patients with distal cholangiocarcinoma and ampullary carcinoma, and 20 healthy individuals. Salivary samples from patients with neoplasia had higher richness in *Prevotella*, *Rothia*, *Veillonella*, *Porphyromonas*, *Actynomyces*, and *Fusobacterium*, while healthy controls had higher abundance of *Streptococcus*. Additionally, PC patients showed enrichment in *Rothia* and *Leptotrichia* compared with cholangiocarcinomas and ampullary carcinomas [55]. A multinational study (Japan, Spain and Germany) conducted by Nagata et al. presented a shotgun metagenomic analysis of fecal and salivary samples collected in treatment-naive PC patients and healthy controls. Specifically, the oral microbiome of PC patients was significantly enriched in *Firmicutes* and *Prevotella* spp., while some *Streptococcus* spp., such as *Streptococcus salivarius*, were depleted. The latter result agrees with the other studies [42,43], although the increase in *P. gingivalis* and the decrease in *Neisseria* spp. were not replicated in this cohort [56]. From the data collection of Nagata et al., Shen et al. investigated the potential to leverage the genomic signatures of antibiotic resistance genes (ARGs) associated with the oral microbiome for the diagnosis of PC. Overall, 3 enriched ARGs were identified in healthy subjects and 21 were significantly elevated in PC patients, suggesting a possible link between antibiotic resistance and PC occurrence. A diagnostic model based on ARG profiles was developed with 79% accuracy (top predictive ARGs: tet(Q), CSP-1 and mefE) [57]. Based on the same dataset, Zeng et al. evaluated the potential role of virulence factors (VFs) encoded by the salivary microbiome of healthy controls and PC patients. VFs are molecules produced by micro-organisms that contribute to their pathogenicity, such as through promoting colonization, tissue invasion, and immune evasion. Specifically, the authors identified three categories of VFs enriched in PC (adherence, exoenzyme and nutritional/metabolic processes) and one enriched in healthy subjects (immune modulation), observing an overall difference in VFs’ composition (*p* = 0.048). Additionally, they developed a diagnostic model based on VF profiles that achieved an accuracy of 0.88, outperforming the model by Nagata et al. (AUC 0.78–0.82), suggesting that the inclusion of VFs could provide a more dynamic and functional perspective of the role of the salivary microbiome in the pathogenesis of neoplasia [58].

### 4.2. Tumor Pathophysiology and Progression

The salivary microbiota has also been shown to play a potential role in tumor pathophysiology and the progression of PC (Table 1). The translocation of bacteria from the oral cavity to pancreatic tissue can be attributed to two mechanisms [59]. Firstly, via gastrointestinal reflux (the oral–gut–pancreas axis), bacteria can pass through the stomach and travel retrograde into the pancreatic duct via the ampulla of Vater. Studies comparing bacterial profiles in duodenal fluid and pancreatic tissue support this pathway [14]. The second is represented by haematogenous dissemination. In individuals with chronic periodontitis or gingival inflammation, ulcerations in the oral epithelium allow bacteria such as *P. gingivalis*, *A. actinomycetemcomitans*, and *F. nucleatum* to enter the bloodstream and, via transient bacteraemia, reach the pancreatic tissue [59].

A case–control study investigated the role of the salivary microbiota in modifying certain pathways favoring carcinogenesis. By analyzing saliva and blood samples from 309 cancer patients (50 with PC) and 745 healthy controls, the authors found that smoking and alcohol consumption were associated with decreased levels of *Prevotella*, *Haemophilus* and *Neisseria* and increased levels of *Streptococcus*, *Abiotrophia* and *Leuconostoc*. These microbial shifts led to altered metabolic output, notably reduced levels of SCFAs and downregulation of free fatty acid receptor 2 (FFAR2) expression. This process was associated with increased serum levels of cytokine concentrations such as tumor necrosis factor alpha-induced protein 8 (TNFAIP8) and interleukin-6/signal transducer and activator of transcription 3 (IL-6/STAT3), thereby promoting chronic inflammation and potentially increasing the risk of cancer onset [32]. As demonstrated by the study by Fan et al., *P. gingivalis*, a known periodontal pathogen, may play a role in PC oncogenesis [22]. Tan et al. compared the salivary and tissue microbiome of 21 patients with PC. The authors demonstrated that *P. gingivalis* is equally present in both microenvironments, and in greater quantities in PC tissue than in the surrounding healthy tissue (*p* < 0.05). Using mouse models, it was demonstrated that this microorganism could translocate from the oral cavity to the pancreas and, by modifying the inflammatory tumor microenvironment (TME), promote carcinogenesis. Specifically, *P. gingivalis* has been shown to downregulate immune marker genes in lymphocyte chemotaxis, defense response to Gram-negative bacteria and cytosis-related genes, while upregulated genes associated with the inflammatory response and neutrophil chemotaxis; furthermore, *P. gingivalis* increases the secretion of neutrophil elastase (NE) from tumor-associated neutrophils to promote PC progression [60].

Another study conducted by McKinley et al. analyzed saliva and pancreatic tumor samples from 29 resectable PC patients with the aim of assessing the potential of bacterial translocation in the process of tumorigenesis. *Veillonella* and *Streptococcus* were constantly found in the oral microbiota of all patients. Notably, these same genera were also highly abundant in tumor tissue, particularly *Veillonella*, which was detected in 92% of tumors. Comparing matched samples, the *Veillonella* strains were frequently found in both saliva and tumor tissue, supporting the hypothesis of a direct oral translocation through the bloodstream. In addition, the authors studied the genome of *Veillonella atypica* (*V. atypica*) to analyze genes belonging to cancer-related pathways. In particular, three pathways (nitrogen metabolism, quorum sensing and biofilm formation, and lipopolysaccharide biosynthesis) have been identified as being involved in tumor progression through the colonization of tumor tissue, co-aggregation with other bacteria, stimulation of chronic inflammation, and modulation of the host immune response [61]. In the study by Uguz et al., the authors also demonstrated how numerous oral bacteria (*Rothia mucilaginosa* e *Veillonella dispar*) remained enriched even in tissue samples from patients with neoplasia, supporting a potential role of oral dysbiosis and bacterial translocation in carcinogenesis [55].

An observational study by Zhu et al. evaluated the gradual shift in the composition of the microbiome from saliva, duodenal fluid, and pancreatic tissue samples among patients with chronic pancreatitis and benign and malignant pancreatic tumors. In saliva samples, *Peptoanaerobacter stomatis* and *Propionibacterium acidifaciens* increase their levels along with disease progression, while others, such as *Fusobacterium mortiferum*, *Lactiplantibacillus plantarum*, and *Loigolactobacillus coryniformis*, showed decreasing trends from chronic pancreatitis to stage IV PC patients. The authors demonstrated that salivary and duodenal microbiota contribute to the composition of pancreatic microbiota during PC progression, using the neutral community model (NCM) [62].

The same group of researchers recently used a NetShift analysis to compare microbial interaction networks in pancreatic tissue, duodenal fluid, and saliva between 63 PC patients and 22 benign controls. In saliva, *Slackia exigua*, *Prevotella dentalis*, *Anaeroglobus geminatus*, and *Propionibacterium acidifaciens* were identified as key microbes driving the development of PC [63].

The study by Matsukawa et al. evaluated the complex network established between salivary, gut and tumor microbiota in PC patients. Notably, *Microbacterium* and *Stenotrophomonas* detected in pancreatic tumor tissue showed a link with fecal and salivary microbes. This finding demonstrates how the salivary microbiota is an active part of a systemic microbial network potentially involved in tumor pathophysiology and progression [64]. Another study exploring bacterial translocation that is potentially involved in PC tumorigenesis was conducted by Chung et al. The researchers collected oral samples (including saliva), as well as pancreatic tissue and other intestinal sites, from 52 subjects with PC and other gastrointestinal conditions. By analyzing amplicon sequence variants (ASVs), it was found that certain bacterial species, such as *F. nucleatum* subsp. *vincentii* and *Gemella morbillorum*, showed high concordance between saliva and pancreatic and intestinal tissues. Moreover, some pathogenic species known to be associated with periodontal disease and cancer, such as *Campylobacter rectus* and *F. nucleatum*, respectively, have been found in clusters of co-occurrence in both saliva and PC tissues [65].

**Figure 2:**
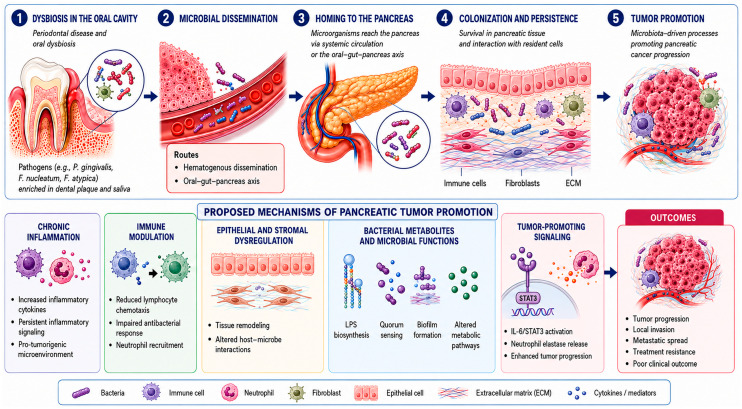
This figure illustrates the mechanisms linking oral dysbiosis (1) to the development of PC (5). Once these microorganisms have colonized pancreatic tissue, they can promote the onset and progression of the tumor through chronic inflammation, immune modulation, epithelial and stromal remodeling, microbial metabolites and the activation of oncogenic signaling pathways, ultimately contributing to tumor growth, invasiveness, metastasis and resistance to treatment. AI-assisted (ChatGPT, OpenAI, available at https://chat.openai.com/ (accessed on 22 May 2026)) schematic illustration, reviewed and validated by the authors.

In conclusion, the various studies cited emphasize that the presence of specific oral bacteria in both saliva and pancreatic tissue reinforces the hypothesis that salivary microbiota may influence pancreatic disease processes. Periodontal disease can lead to persistent immune activation, resulting in the release of inflammatory mediators responsible for a systemic inflammatory response and, consequently, the carcinogenic process [60]. Therefore, periodontal pathogens such as *P. gingivalis* and *F. nucleatum* may play a key role in the link between oral dysbiosis and the development of PC (Figure 1 and Figure 2).

**Table 1:** The Salivary Microbiota in Pancreatic Carcinoma.

Authors [References]	Setting	Population	Results	Methodology	Proposed Biological Mechanisms
Farrell et al. [42]	Screening	PC (resectable/borderline resectable) pts vs. healthy controls	- *N. elongata* and *S. mitis* are reduced in PC pts. - *Granulicatella adiacens* is increased in PC pts.	qPCR	LPS–TLR4–NF-κB signaling
Tavanaeian et al. [43]	Screening	PC pts vs. healthy controls	- *N. elongata* is reduced in PC pts. - *Granulicatella adiacens* is increased in PC pts.	16S rRNA	NA
Olson et al. [47]	Screening and diagnosis	PC pts vs. IPMNs pts vs. healthy controls	- *Firmicutes* levels are higher in PC pts. - *Proteobacteria* levels are higher in controls. - *Neisseria elongata* is reduced in PC pts.	16S rRNA	NA
Lin et al. [48]	Screening and diagnosis	PC pts vs. pancreatitis vs. healthy controls	- *Bacteroides* levels are higher in PC. - *Corynebacterium* and *Aggregatibacter* are lower in PC.	16S rRNA	NA
Torres et al. [49]	Screening and diagnosis	PC pts vs. other diseases (including cancer) pts vs. healthy controls	- *Firmicutes* levels are higher in PC pts. - *Proteobacteria* levels are higher in controls. - *Bacteroides* levels are higher in PC. - *Neisseria* and *Aggregatibacter* are lower in PC.	16S rRNA	NA
Sun et al. [50]	Screening and diagnosis	PC pts vs. pancreatitis pts vs. healthy controls	- *Firmicutes*, *Bacteroides* and *Fusobacteria* levels are higher in PC pts. - *Proteobacteria* levels are higher in controls.	16S rRNA	FadA–β-catenin signaling
Fan et al. [22]	Screening, Tumor pathophysiology	PC pts vs. healthy controls	- *Fusobacteria* was associated with reduced risk of PC. - *P. gingivalis* and *A. actinomycetemcomitans* were associated with increased risk of PC.	16S rRNA	Immune evasion and TLR signaling activation
Nouri et al. [32]	Screening, Tumor pathophysiology	OC, HNC, PC, GC vs. healthy controls	- *Haemophilus, Prevotella* and *Neisseria* are associated with decreased odds of PC. - *Streptococcus*, *Leuconostoc* and *Abiotrophia* are associated with increased odds of PC. - Correlation between oral microbiome in cancer pts, reduced levels of SCFAs and FFAR2, and increased levels of TNFAIP8 and IL-6/STAT3.	16S rRNA	SCFA–FFAR2–TNFAIP8–IL-6/STAT3 signaling axis
Hesami et al. [51]	Screening	PC pts vs. healthy controls	- *P. gingivalis* and *A. actinomycetemcomitans* were associated with increased risk of PC.	qPCR	NA
Vogtmann et al. [52]	Screening	PC pts vs. healthy controls	- *Haemophilus* is associated with decreased odds of PC. - *Enterobacteriaceae*, *Lachnospiraceae G7*, *Bacteroidaceae*, and *Staphylococcaceae* are associated with increased odds of PC.	16S rRNA	NA
Wei et al. [53]	Screening	PC pts vs. healthy controls	- *Veillonella* and *Neisseria* are associated with decreased odds of PC. - *Streptococcus* and *Leptotrichina* are associated with increased odds of PC. - Correlation between bacterial species and clinical symptoms.	16S rRNA	NA
Oh et al. [54]	Screening	PC vs. EC vs. GC vs. BC pts vs. healthy controls	- Difference in microbiome composition between EC and GC vs. PC (*p* = 0.001) and BC vs. PC (*p* = 0.013). - *Akkermansia*, *Para bacteroides*, *Blautia*, *Collinsella*, *Escherichia-Shigella*, *Subdoligranulum*, and *Fusicatenibacter* are increased in PC pts vs. controls. - *Actinomyces*, *Neisseria*, and *Stomatobaculum* are reduced in PC pts vs. controls.	16S rRNA	NA
Uguz et al. [55]	Screening, Tumor pathophysiology	PC pts vs. DC/AC pts vs. healthy controls	- *Prevotella*, *Rothia*, *Veillonella*, *Porphyromonas*, *Actynomyces*, and *Fusobacterium* are higher in PC/DC/AC pts. - *Streptococcus* is higher in healthy controls. - Several oral bacteria (*Rothia mucilaginosa* and *Veillonella dispar*) remained enriched even in tissue samples, suggesting how bacterial translocation triggers tumorigenesis.	16S rRNA	NA
Nagata et al. [56]	Screening, Prognosis	PC pts vs. healthy controls	- *Firmicutes* and *Prevotella* levels are higher in PC pts. - *Streptococcus* (*S salivarius*, *S thermophilus*, *S australis*) levels are lower in PC. - *Capnocytophaga* sp. was associated with favorable prognosis. - *Streptococcus vestibularis* and *Neisseria bacilliformis* were correlated with worse prognosis.	Shotgun	NA
Shen et al. [57]	Screening, Tumor pathophysiology, Influence on therapy	PC pts vs. healthy controls	- Increased number of ARGs in PC pts.	Shotgun	NA
Zeng et al. [58]	Screening, Tumor pathophysiology and progression, Prognosis	PC pts vs. healthy controls	- 3 VFs (adherence, exoenzyme and nutritional/metabolic processes) are enriched in PC pts.	Shotgun	NA
Tan et al. [60]	Tumor pathophysiology and progression	PC pts	- *P. gingivalis* induces pro-inflammatory TME with an elevation of NE, promoting PC progression.	16S rRNA	TAN/NE pathway
McKinley et al. [61]	Tumor pathophysiology and progression	PC pts	- *V. atypica* can translocate from the mouth to pancreatic tissue in PC pts, contributing to tumorigenesis and progression.	16S rRNA	NA
Zhu et al. [62]	Tumor pathophysiology and progression, Prognosis and influence on therapy	PC pts and other pts (pancreatic benign tumors and chronic pancreatitis)	- *Peptoanaerobacter stomatis* and *Propionibacterium acidifaciens* levels increase along with disease progression. - *Chryseobacterium montanum*, *Comamonas testosteroni* and *Methylobacillus flagellates* are enriched in stable/progression disease pts. - *P. gingivalis*, *Porphyromonas endodontalis* and *Filifactor alocis* are enriched STS pts.	16S rRNA	NA
Zhu et al. [63]	Tumor pathophysiology, Diagnosis	PC pts vs. other pts (neuroendocrine tumor, autoimmune pancreatitis, etc.)	- *Slackia exigua*, *Prevotella dentalis*, *Anaeroglobus geminatus*, and *Propionibacterium acidifaciens* have been identified as key microbial drivers in the development of PC. - Salivary model discerned PC pts from pancreatic benign tumor pts with an accuracy of 0.963 AUC.	16S rRNA	NA
Matsukawa et al. [64]	Tumor pathophysiology	PC pts	- There is a complex network between the gut, salivary and tumor microbiota.	Shotgun	NA
Chung et al. [65]	Tumor pathophysiology	PC pts, BC pts, other pancreatic and gastrointestinal diseases	- *F. nucleatum* subsp. *vincentii* and *Gemella morbillorum* showed high concordance between saliva and pancreatic and intestinal tissues.	16S rRNA	NA
Archibugi et al. [66]	Prognosis	PC pts	- *Fusobacterium* and *Tannerella* higher are associated with better prognosis. - *Streptococcus* and *Actinobacillus* are associated with worse prognosis.	16S rRNA	NA

Abbreviations: PC: pancreatic cancer; IPMNs: Intraductal Papillary Mucinous Neoplasms; pts: patients; *N. elongata*: *Neisseria elongata*; *S. mitis*: *Streptococcus mitis*; *P. gingivalis*: *Porphyromonas gingivalis*; *A. actinomycetemcomitans*: *Aggregatibacter actinomycetemcomitans*; *F. nucleatum*: *Fusobacterium nucleatum*; *V. atypica*: *Veillonella atypica*; OC: oral cancer; HNC: head and neck cancer; GC: gastric cancer; 16S rRNA: 16S ribosomal ribonucleic acid; TLR4: Toll-Like Receptor 4; NF-κB: Nuclear Factor kappa-light-chain-enhancer of activated B cells; FadA: Fusobacterium adhesin A; SCFAs: short-chain fatty acid; FFAR2: free fatty acid receptor 2; TNFAIP8: tumor necrosis factor alpha induced protein 8; IL-6: interleukin-6; STAT3: signal transducer and activator of transcription 3; EC: esophageal cancer; BC: biliary tract cancer; DC/AC: periampullary cancers; ARGs: antibiotic resistance genes; VFs: virulence factors; TME: tumor microenvironment; TAN: Tumor-Associated Neutrophils; NE: neutrophils elastase; STS: short-term survival; AUC: area under the curve; NA: not available.

### 4.3. Prognosis Implications and Influence on Therapy

The salivary microbiota has also been shown to play a role in terms of prognosis and to influence the efficacy of cancer treatment, although the evidence is less than that obtained in the two previously presented settings (Table 1).

In a recent prospective observational study, the association between microbiome signatures and overall survival (OS) was investigated in 23 patients with pancreatic cancer. Higher salivary phylogenetic diversity was associated with longer survival (aHR = 0.19, *p* = 0.001). Furthermore, numerous genera are independently associated with OS in a multivariate analysis adjusted for age and disease stage. In particular, high levels of *Fusobacterium* and *Tannerella* are associated with a better prognosis, while *Streptococcus* and *Actinobacillus* are associated with poorer survival [66].

In the multinational metagenomic study by Nagata et al., the authors used the Cox regression model with LASSO feature selection to identify 10 microbial species strongly associated with the prognosis of patients with PC in both the gut and salivary microbiota. Specifically, unknown *Capnocytophaga* sp. in the saliva was associated with favorable prognosis (*p* < 0.05) while *Streptococcus vestibularis* and *Neisseria bacilliformis* were correlated with worse prognosis (*p* = 0.05 and *p* = 0.02, respectively). Also, through performing a time-to-event analysis, it was shown that high abundance of species such as *Streptococcus pneumoniae* was negatively associated with the probability of disease progression. A subgroup analysis was also conducted in a study considering early- and advanced-stage patients, patients treated with adjuvant and neoadjuvant chemotherapy, and patients receiving or not receiving FOLFIRINOX/nab-paclitaxel. Of the 10 microbial species, 7 gut and salivary species were found to be associated with survival in at least one group; *Neisseria bacilliformis* in the saliva, detected in four groups, was found to be a potential and reliable prognostic marker [56]. In the study by Zhu et al., differences in the microbiome between chemotherapy responders and non-responders were studied; specifically, in the saliva samples, *Chryseobacterium montanum*, *Comamonas testosteroni*, and *Methylobacillus flagellates* were enriched in stable/disease-progression patients compared with complete-response/partial response patients. This finding could suggest that the composition of the microbiota may correlate with the efficacy of treatment. In terms of prognosis, *P. gingivalis*, *Porphyromonas endodontalis* and *Filifactor alocis*, which intensified from chronic pancreatitis to stage IV PC in pancreatic tissue, were also reported to be enriched in the saliva of patients with short-term survival compared to those with long-term survival, while *Fermentimonas caenicola* was decreased [62].

Shen et al. [57] and Zeng et al. [58], in the final part of their articles, speculate on the potential role of ARG diversity and VFs expression in prognosis and the influence on cancer treatments, respectively. In the first study, they hypothesize that higher ARG diversity in the microbiota of PC patients could have implications for therapy and intensity of dose, as this could predispose patients to a higher frequency and severity of infections resistant to standard antibiotic treatments [57]. In the second study, the authors claim that VFs could be useful not only from a prognostic point of view but also in monitoring disease progression and assessing response to oncological therapies [58].

However, the biological significance linking the various microbial taxa to oncological outcomes remains unclear. Indeed, these microorganisms may act as genuine biological modulators of prognosis and treatment response or, alternatively, as surrogate markers of disease-related processes, such as metabolic alterations, systemic inflammation or immune competence. Only mechanistic evidence will eventually be able to validate the prognostic and predictive relevance of these microbial signatures.

## 5. Conclusions

Recent evidence suggests that salivary and oral microbiota may play a significant role in the pathogenesis of PC. Specific alterations in microbial composition and oral infections seem to contribute to a pro-inflammatory and immunomodulatory microenvironment, which can encourage tumor initiation and progression. Concurrently, characteristics of the oral/salivary microbiota are emerging as potential, easily accessible and non-invasive biomarkers for identifying individuals at risk and tracking the illness. However, in order to understand the real contribution that the salivary and oral microbiota can make in the clinical management of PC and to establish the existence of causal links, well-designed large-scale longitudinal studies would be required. In addition, standardizing the steps of saliva collection, storage and processing would represent a critical issue to ensure the consistency and reproducibility of observations. At the same time, future studies in this context should take into account confounding factors such as smoking, periodontal disease, antibiotic exposure, diet, and comorbidities, which could influence salivary microbiota profiles [59]. A promising direction involves the integration of microbiome profiling with complementary omics technologies, including metabolomics, transcriptomics and proteomics. Multi-omics approaches could help shed further light on host–microbiome interactions and their role in the pathogenesis of PC. Furthermore, future investigations should examine the influence of the salivary microbiota on chemotherapy, immunotherapy and treatment-related toxicity in pancreatic cancer, with the aim of identifying microbial signatures with a proven prognostic and predictive role.

Salivary microbiota could therefore serve as a complementary tool in the management of pancreatic cancer; however, further validation is required through comparative studies with tumor markers and imaging approaches. In the end, introducing the investigation of oral/salivary microbiota in the context of PC may provide fresh insights for more individualized preventive, diagnostic, and therapeutic approaches, potentially leading to improvements in a prognosis that is currently among the most unfavorable in oncology.

## Data Availability

No new data were created or analyzed in this study. Data sharing is not applicable to this article.

## Abbreviations

The following abbreviations are used in this manuscript:

PC	Pancreatic cancer
*N. elongata*	*Neisseria elongata*
*S. mitis*	*Streptococcus mitis*
*P. gingivalis*	*Porphyromonas gingivalis*
PDAC	Pancreatic ductal adenocarcinoma
BRCA2	Breast Cancer Type 2 Susceptibility Gene
CDKN2A	Cyclin-Dependent Kinase Inhibitor 2°
MSH2	MutS Homolog 2
MLH1	MutL Homolog 1
APC	Adenomatous Polyposis Coli
STK11	Serine/Threonine Kinase 11
LTKB1	Liver Kinase B1
16S rRNA	16S ribosomal ribonucleic acid
SCFAs	short-chain fatty acids
LPS	lipopolysaccharide
TLR4	Toll-Like Receptor 4
NF-κB	Nuclear Factor kappa-light-chain-enhancer of activated B cells
FadA	Fusobacterium adhesin A
TNF-α	tumor necrosis factor alpha
PanIN	Pancreatic Intraepithelial Neoplasia
*C. albicans*	*Candida albicans*
*A. actinomycetemcomitans*	*Aggregatibacter actinomycetemcomitans*
*F. nucleatum*	*Fusobacterium nucleatum*
NE	neutrophil elastase
IPMNs	intraductal papillary mucinous neoplasms
HOMIM	Human Oral Microbe Identification Microarray
qPCR	quantitative Polymerase Chain Reaction
GC	gastric cancer
EC	esophageal
BC	biliary cancer
ARGs	antibiotic resistance genes
VFs	virulence factors
FFAR2	free fatty acid receptor 2
TNFAIP8	tumor necrosis factor alpha-induced protein 8
IL-6	interleukin-6
STAT3	signal transducer and activator of transcription 3
TAN	Tumor-Associated Neutrophils
TME	tumor microenvironment
*V. atypica*	*Veillonella atypica*
NCM	neutral community model
ASVs	amplicon sequence variants
OS	overall survival
OC	oral cancer
HNC	head and neck cancer
DC/AC	periampullary cancers
STS	short-term survival
AUC	area under the curve
